# Following damage, the majority of bone marrow-derived airway cells express an epithelial marker

**DOI:** 10.1186/1465-9921-7-145

**Published:** 2006-12-19

**Authors:** Heather MacPherson, Pamela A Keir, Carol J Edwards, Sheila Webb, Julia R Dorin

**Affiliations:** 1 MRC Human Genetics Unit, Western General Hospital, Crewe Road South, Edinburgh EH4 2XU, UK

## Abstract

**Background:**

Adult-derived bone marrow stem cells are capable of reconstituting the haematopoietic system. However there is ongoing debate in the literature as to whether bone marrow derived cells have the ability to populate other tissues and express tissue specific markers. The airway has been an organ of major interest and was one of the first where this was demonstrated. We have previously demonstrated that the mouse airway can be repopulated by side population bone marrow transplanted cells. Here we investigate the frequency and phenotypic nature of these bone marrow derived cells.

**Methods:**

Female mice were engrafted with male whole bone marrow or side population (SP) cells and subjected to detergent-induced damage after 3 months. Donor cells were identified by Y chromosome fluorescence in situ hybridisation and their phenotype was assessed by immunohistochemistry on the same sections. Slides were visualised by a combination of widefield and deconvolved microscopy and whole cells were analysed on cytospin preparations.

**Results:**

The frequencies of engraftment of male cells in the airway of mice that show this (9/10), range from 1.0 – 1.6% with whole marrow and 0.6 – 1.5% with SP cells. Undamaged controls have only between 0.1 and 0.2% male cells in the trachea. By widefield microscopy analysis we find 60.2% (53/88) of male donor derived cells express cytokeratins as a marker of epithelial cells. These results were reinforced using deconvolved microscopy and scored by two independent investigators. In addition cytospin analysis of cells dissociated from the damaged trachea of engrafted mice also reveals donor derived Y chromosome positive cells that are immunopositive for cytokeratin. Using cytokeratin and the universal haematopoietic marker CD45 immunohistochemistry, we find the donor derived cells fall into four phenotypic classes. We do not detect cytokeratin positive cells in whole bone marrow using cytokeratin immunostaining and we do not detect any cytokeratin mRNA in SP or bone marrow samples by RT-PCR.

**Conclusion:**

The appearance of bone marrow derived cells in the tracheal epithelium is enriched by detergent-induced tissue damage and the majority of these cells express an epithelial marker. The cytokeratin positive donor derived cells in the tracheal epithelium are not present in the injected donor cells and must have acquired this novel phenotype *in vivo*.

## Background

The potential of adult-derived bone marrow cells being localised to the airway is an attractive, novel therapeutic approach for pulmonary repair. Much scientific debate has centred on the ability of bone marrow-derived cells to engraft into non-haematopoietic tissues and assume an epithelial phenotype. The airway has been demonstrated to be repopulated by bone marrow transplanted cells[1,2]. There have been reports of extremely high levels of donor-derived cells[3,4] but subsequent reports from the same groups have either reduced this figure[2] or refuted their own claims[5]. Some of the confusions are undoubtedly due to the necessity for a particular type of damage (but this is not well characterised) and analysis techniques[6].

Often the detection of donor bone marrow-derived cells relies on the expression of a transgenic marker present only in that population. However, we found in the airway that expression of the β-*galactosidase *transgene (present in the male Rosa 26 donor cells) was not apparent in cells that could be determined as donor on the basis of Y chromosome fluorescence in situ hybridisation (FISH). This may be a result of donor gene inactivation, down-regulation or elimination [7]. Thus experiments that rely on donor contribution by virtue of transgene expression could underestimate the number of cells[5,8]. The reverse is also true, as the colorimetric β-*galactosidase *substrate 5-bromo-4-chloro-3-indolyl-beta-D-galactopyranoside (X-gal) in adult tissue can be subject to false positive staining.

Detection of cells coincident for an immunohistochemical signal using serial sections is subject to error. It has also been questioned whether the use of conventional widefield microscopy on the same section is able to resolve overlapping or juxtapositioned cells[8]. This could lead to erroneous identification of single cells with co-localised markers rather than two independent cells. The use of confocal or deconvolution microscopy on the same section allows discrimination of adjacent cells and a true determination of colocalisation of markers.

The engraftment of bone marrow cells into another tissue type has been described as transdifferentiation but recent work has suggested that fusion may be a more likely explanation. Bone marrow-derived cells in the liver and kidney have been shown to have arisen as a consequence of fusion [7,9-12]. However Harris et al found no evidence of fusion in lung, skin and liver [5] while Brittan [13] et al found no evidence in the epidermis.

We have previously demonstrated that bone marrow-derived side population (SP) cells contribute to the respiratory tract of mice following engraftment of the haematopoietic system and tracheal damage [1]. Here we extend this study to reveal that whole bone marrow donor cells also contribute to the tracheal epithelium following damage but without damage the number of donor cells is 10 fold less. In the four out of five animals transplanted with SP cells that showed airway engraftment, we determine which donor cells express a blood cell marker and which express the epithelial cell marker cytokeratin. We use Y chromosome FISH to identify donor-derived cells and deconvolved imaging to confirm colocalisation of these cells with the epithelial marker cytokeratin. We find that the majority of donor-derived cells express cytokeratin, and some of these also express the CD45 haematopoietic marker. A proportion of cells are consistent with being inflammatory cells in that they only express CD45. Some cells express neither haematopoietic nor epithelial cell markers. We demonstrate that the donor cell population does not express cytokeratin prior to injection and so the cells in the epithelium have acquired a novel phenotype *in vivo*.

## Materials and methods

### Mouse strains

Male *ROSA26 *mice of 6–10 weeks of age and backcrossed for 5 generations to CBA/Ca, were bred in house and used to isolate bone marrow. CBA/Ca female mice aged 8–12 weeks obtained from Charles River UK Ltd (England, UK) were used as bone marrow recipients.

### Bone marrow preparation

Bone marrow was extracted from the femurs and tibias of mice and a single cell suspension prepared by passing the bone marrow through a 21-gauge needle after which the cells were pelleted by centrifugation and resuspended at 10^6 ^cells/ml in DMEM that contained 2% fetal calf serum (FCS) and 10 mM Hepes (Gibco-BRL, Paisley, UK).

### SP cell isolation

SP cells were isolated from the bone marrow according to published methods [14]. Briefly, bone marrow was resuspended at 10^6 ^cells/ml in prewarmed DMEM as outlined above. Hoechst 33342 (Bis-Benzimide) (Sigma Aldrich, Dorset, UK) was added to the cells at a final concentration of 5 μg/ml and the cells incubated at 37°C for 90 minutes. After this time, the cells were pelleted and resuspended in ice cold HBSS containing 2% FCS and 10 mM Hepes (Gibco-BRL, Paisley, UK) and maintained at 4°C for analysis by flow cytometry.

### Tracheal cell isolation

Tracheas were collected into PBS and digested for 1 hour at 37°C in Ca^2+^, Mg^2+ ^free media containing 1.4 mg/ml Pronase (Roche, Lewes, UK) and 0.1 mg/ml Dnase1 (Sigma, Poole, UK). FCS was added to halt the reaction and the husks washed twice with 2% FCS in PBS then discarded. Cells were pelleted and resuspended at 10^5 ^cells/ml in 2% FCS in PBS. For cytospin preparation cells were then applied to slides by centrifugation at 10^4 ^cells/slide using a Thermo-Shandon Cytospin4 (Pittsburgh, PA) at 800 rpm for 7 minutes. They were then fixed for 10 minutes in 4% paraformaldehyde (Sigma, Poole, UK) at room temperature, washed twice in PBS for 5 minutes each, and air dried before storage at -80°C.

### Flow cytometry

Analysis and sorting was performed using a FACSVantage, equipped with a Coherent INNOVA Enterprise II laser (Becton and Dickinson, Oxford, UK) and performed as originally described[14]. Briefly, the Hoechst dye was excited at 350 nm and its fluorescence emission was collected with a 424/44 band pass (BP) filter (Hoechst blue) and a 675/20 BP filter (Hoechst red). A 610 SP was used to separate the blue and red emissions. Propidium Iodide (PI) (Sigma Aldrich, Dorset, UK) was added to the cells prior to sorting at a final concentration of 2 μg/ml to distinguish and exclude dead cells in the bone marrow population. Sort gates were set as defined by Goodell[14]. After acquiring 1 × 10^5 ^live cells, the SP gate could be clearly defined. The SP cells were then sorted into polypropylene tubes (BD Labware Europe, Combourg, France) containing 100% FCS and an aliquot re-analysed to check cell sort purity.

### Irradiation and cell infusion

CBA/Ca female mice (8–12 weeks) were lethally irradiated with 1050 rads delivered from a GammaCell 40E (MDS Nordian, Fleuvus, Belgium) with a Cesium 137 source at a dose-rate of 114 Rads/minute. Following irradiation they received a single tail-vein infusion of 1 × 10^4 ^bone marrow-derived SP cells. In experiments where whole bone marrow was used 1 × 10^7 ^cells were injected. The animals were housed under specific pathogen free (SPF) conditions in IVCs for 3 months. If the tracheas were damaged, this was done by instillation with 10 μl 2% polidocanol (Sigma, Poole, UK) (SP 1–5) or polidocinol plus 1 mg/ml E.coli lipoplysaccharide (Sigma Poole, UK) applied intranasally on day 2. All animals were harvested 7 days after the polydocinol instillation.

Experimental protocols involving animals were carried out in accordance with permits and guidelines issued by the MRC Ethical Review Committee and the United Kingdom Home Office.

### Immunohistochemistry and histology of sections

Tracheas were removed from the appropriate animals and fixed in 4% paraformaldehyde (PFA), processed to paraffin wax blocks and cut to 7 μM sections by microtome. Sections were then deparaffinised in xylene and hydrated through an alcohol series before mounting for viewing, carrying out immunostaining or for FISH analysis.

CD45 staining was performed before FISH. Slides were dewaxed by washing 3 times in xylene for 5 minutes before rehydration through an ethanol series (100%, 90%, 70%, 50%, 30%) and then placed into water. The slides were then microwaved for 20 minutes in 0.1 M citrate buffer ph 6.0. Slides were rinsed in PBS and then blocked with 10% goat serum (Sigma, Poole, UK) for 1 hour at RT after which time the slides were washed in 3 times in PBS. The slides were incubated with rat anti-mouse CD45 antibody (1 in 10) (BD Pharmingen, Oxford, UK) overnight at 4°C. The slides were washed as before and then incubated with CY3 labelled goat anti rat secondary antibody (1:50) (Jackson ImmunoResearch Laboratories, USA) for 2 hours at RT then washed and mounted with Vectashield (Vector Laboratories, Peterborough UK) containing 1 μg/ml 4,6-diaminidino-2-phenylindole (DAPI) counterstain.

Cytokeratin staining was performed after FISH. Slides were blocked with 1% IgG (Sigma, Poole, UK) diluted in 5% donkey serum (Sigma, Poole, UK) for 20 minutes at RT, washed 3 times in PBS and then blocked again in 5% donkey serum, 2% BSA in PBST (0.1% Tween in PBS) for 30 minutes at RT. The slides were washed as before and incubated with mouse pan cytokeratin ascites fluid (Sigma, Poole, UK) (1:20) overnight at 4°C. The slides were washed and then incubated with FITC labelled donkey anti-mouse F(ab) fragment (1:50) (Jackson ImmunoResearch Laboratories, USA) secondary antibody for 2 hours at RT then washed and mounted with Vectashield containing 1 μg/ml DAPI. Negative controls using irrelevant isotype antibodies or missing out the primary antibody were carried out in parallel with every experiment. Spleen was used as a positive control for CD45 as detailed on IHC World online information center for immunohistochemistry [15]. Sections were then stained with hematoxylin and metanil yellow according to standard protocols.

### Immunostaining of cytospun cells

Cytokeratin staining was performed before FISH. Briefly, slides were washed three times in PBS, and then blocked for 1 hour at RT in 10% goat serum (Sigma, Poole, UK). The cytokeratin (1:1500) (DAKO Corporation, Carpinteria, CA) antibody was then applied and the slides incubated for 1 hour at RT. After this time they were washed once in 0.1% Tween-20 in PBS for 5 minutes followed by two washes in PBS. They were then incubated in the FITC-conjugated cytokeratin secondary antibody (Jackson ImmunoResearch Laboratories, Peterborogh, UK) for 1 hour at RT. The slides were then washed as above before mounting in Vectashield containing 1 μg/ml DAPI. Cytospins were stained with hematoxylin and eosin (H and E) according to a standard procedure.

### FISH analysis for sections

Slides were dewaxed and rehydrated as before. The slides were then microwaved for 20 minutes in 0.1 M citrate buffer ph 6.0 after which time they were denatured for 3 minutes in 70% formamide/2 × SSC, plunged into ice cold 70% ethanol and dehydrated through an alcohol series and air-dried.

Y chromosome FISH was carried out using a biotinylated whole chromosome Y paint (Cambio, Cambridge, UK) according to the manufacturers instructions. Briefly, the probe was hybridised to the section overnight at 37°C. Slides were then washed 4 times for 3 minutes in 2 × SSC at 45°C then incubated for 30 minutes at 37°C with avidin Texas Red (Vector Laboratories, Peterborough UK). This was followed by washing 3 times for 2 minutes in 4 × SSC, 0.1% Tween20 at 37°C after which time biotinylated anti avidin (Vector Laboratories, Peterborough UK) was added and the slides incubated further for 30 minutes. The slides were then washed as above and avidin Texas Red added to the slides for a further 30 minutes incubation at 37°C. After washing the slides as before, they were mounted in Vectashield containing 1 μg/ml DAPI. Male sections were taken through as positive controls and female sections as negatives. Perinuclear nuclear signals consistent with the Y chromosome were only observed on the male sections and never on the female slides.

### FISH analysis for cytospins

FISH was carried out as above with the omission of the dewax/rehydration step. Microwave incubation in citrate buffer was for 30 minutes. 93% of tracheal cells from control male mice were positive (930 of 1000 cells counted) and 0.1% of cells from female mice (1 cell in 1000 cells counted) gave a false positive signal.

### RNA extraction and PCR

Total ribonucleic acid (RNA) was extracted from cells using the method of Chomczynski and Sacchi [16]. Cell pellets were suspended in 1 ml of RNAzol (Biogenesis, Dorset, U.K.) and placed in a 2 ml RNAse free tube (Sarstedt, Leicester, U.K.) into which 1/10^th ^volume of chloroform was added. The solution was vortexed and left on ice for 5 mins. The solution was then centrifuged at 10,000 rpm for 15 mins and the upper aqueous layer removed into a fresh tube and an equal volume of ice-cold isopropanol added. The solution was mixed and left at -20°C for 30 mins. The RNA was then precipitated by centrifugation at 10,000 g for 20 mins. The resulting RNA pellet was washed twice with 75% ice cold ethanol (2 × 5 mins 10,000 rpm spins) and resuspended in 20–100 μl of RNAse free water and the concentration and purity of the RNA determined by spectrophotometry (GeneQuant II, Pharmacia Biotechnology, St Albans, U.K.).

### Preparation of cDNA

Complementary DNA (cDNA) was made by the process of reverse transcription using a cDNA synthesis kit (Roche Applied Science). Briefly, 1 μg of RNA in a volume of 8.2 μl was reverse transcribed by mixing with the following components, 2 μl oligo dT primer (0.8 μg/μl), 2 μl reaction buffer (x10), 2 μl dNTP mix (40 mM), 4 μl 25 mM MgCl_2_, 1 μl RNase Inhibitor (50U/μl) and 0.8 μl reverse transcriptase (200u/μl). The reaction was carried out at 25°C for 10 mins and then at 42°C for 60 mins. The tube was then placed at 95°C for 5 mins after which time the cDNA was used for PCR.

### Polymerase chain reaction

Following RNA extraction, cDNAs were prepared from 10^5 ^bone marrow cells or SP cells or non-SP FACS cells or tracheal cells prepared from adult mouse trachea by digestion as previously described and subjected to PCR[17]. cDNAs were amplified using sequence specific mouse primers. Sequences of the primer pairs were as follows (all written 5'-3' with forward primer first): *Actin *(Product size 450 bp) GGCCCAGAGCAAGAGAGGTATCC and ACGCACGATTTCCCTCTCAGC; *CD45 *(Product size 194 bp) CCTGCTCCTCAAACTTCGAC and GACACCTCTGCTGCCTTAGC; Cytokeratin 19 (Product size 316 bp) AAGACCATCGAGGACTTGC and AATCCACCTCCACACTGACC. The primers were used at a final concentration of 1 μM each in the PCR reaction, which were carried out under standard conditions. The thermal cycling protocol for actin and CD45 comprised an initial denaturation step at 94°C for 4 minutes followed by 43 cycles of 94°C for 1 minute, 60°C for 1 minute and 72°C for 1 minute. The final cycle consisted of a re-annealing at 72°C for 10 minutes. A similar protocol was used for cytokeratin 19 amplification except that the annealing temperature was 57°C for 45 seconds. PCR products were visualised on ethidium bromide stained 2% agarose gels using a Herolab Easy RH-3 system (Scotlab, Coatbridge, UK).

### Image analysis

Slides were visualised using a Zeiss Axioplan 2 microscope (Carl Zeiss UK, Welwyn Garden City, UK) equipped with a Ludl filter wheel (Ludl Electronic Products, Hawthorne, NY) and Chroma 83000 triple bandpass filter set (Chroma Technology Corp., Rockingham, VT). Grayscale images were collected with a Coolsnap HQ cooled CCD camera (Roper Scientific, Tucsan, AZ). In-house scripts written for IPLab (Scanalytics Corp.,Fairfax VA) were employed for image capture and image processing. Slides were also visualised using an Olympus 1 × 71 microscope equipped with a ×60 lens. Images were captured using 0.2 μM optical sections and captured using DeltaVision SoftWorx software set at Bin 1 × 1. Further analysis was performed using Imaris software.

## Results

### Donor cell engraftment in the bone marrow

Female mice were subjected to lethal irradiation and rescued by intravenous delivery of either 1 × 10^4 ^SP cells isolated from male ROSA26 bone marrow or with whole marrow (1 × 10^7 ^cells). SP cells are a subset (~0.1%) of bone marrow cells that can be isolated by their ability to exclude the DNA-binding dye Hoescht 33342[14]. They are potent haematopoietic stem cells and even a single SP cell is capable of reconstituting the haematopoietic system[14,18]. Three months after the irradiation, the animals were subjected to detergent-induced epithelial cell stripping of the trachea and sacrificed one week later. Control transplanted animals not subjected to epithelial stripping were also analysed. The level of haematopietic engraftement in these mice was assessed by incubation of the marrow at sacrifice with the non-fluorescent substrate fluorescein Di-(β-D-galactopyranoside) (FDG). Donor cells from Rosa26 mice ubiquitously express the β-galactosidase gene product and so will convert FDG to a fluorescent product which allows FACS analysis to be used to determine the level of haematopoietic re-engraftment. Animals given SP cells all displayed levels of haematopoetic re-engraftment in their marrow equal to or greater than the control Rosa26[1]. Results for the whole bone marrow transplantations are given in additional file 1: FDG conversion by bone marrow and peripheral blood cells from irradiated CBA and whole Rosa 26 bone marrow transplanted mice after 3 months.

marrow reconstitution after 3 months. The percentage of FDG converting cells in both the lymphoid and granulocytic populations are given in both marrow and peripheral blood. Re-engraftment of the undamaged series CW1-4 at 3 months was equivalent to that observed for the SP transplanted animals [1] i.e. not significantly different from the Rosa 26 control. Re-engraftment of the W1-5 was between 40–50% of the Rosa 26 control in the marrow but showed high level of FDG conversion in the peripheral blood fractions.

### Donor cell engraftment in the tracheal epithelium

Y chromosome FISH was then used to determine the number of Y chromosome positive (donor) cells present in the tracheal epithelium of these animals. In sections from female mice, no false positive signal is seen with the Y probe. Only 87% of male trachea probed using this protocol show a positive FISH signal in the nucleus and this under-representation is probably due to the chromosome being lost in the sectioning. We do not correct for this underestimation of male positive cells. In the whole marrow transplanted animals, where there is no tracheal damage (CW1-4) we see no significant donor-cell contribution to the epithelial layer (table 1). Only 0.14% of cells (+/- 0.002) give a Y chromosome FISH positive signal. In the whole marrow transplanted animals, where there is tracheal damage (W1–W5), there is evidence of donor cells in all of the treated mice, mean of 1.25 % of cells (+/- 0.06) in the epithelial layer of the trachea following damage. This is significantly different to the untreated controls (p > 0.01) and not significantly different to the numbers seen for the SP experiment. Sections from the trachea of the SP mice previously reported, revealed 4 out of the 5 mice had Y chromosome positive cells[1]. We have extended the analysis previously done on the SP engrafted mice from 8,056 total cells counted to 35,363 and the number of cells positive for the Y chromosome in the four positive animals remains around 1% (0.94% +/-0.39). Table 1 reveals the spread of Y chromosome positive cells in all the five animals subjected to analysis. Animal SP2 has no Y chromosome positive cells detectable in the cells lining the trachea. This is most likely because the detergent delivery was less successful than in the other animals. The Y chromosome FISH was repeated three times on sections from this animal and each time this no male cells were observed. The level of re-engraftment to the bone marrow of SP2 was similar to the other animals and equivalent to the Rosa26 control [1]. The level of engraftment in the trachea in the positive animals SP1,3,4,5, varied from 0.58% to 1.49%. Overall 350 positive cells were scored out of 34,082 DAPI stained cells in these animals (table 1).

**Table 1:** Contribution of Y chromosome bone marrow SP cells to female recipient tracheas.

**Mouse**	**Y Chromosome +'ve**	**Total DAPI Nuclei**	**% Y Chromosome +'ve**
CW1	2	2065	0.09
CW2	3	1738	0.17
CW3	2	1818	0.11
CW4	4	2098	0.19
W1	24	2356	1.02
W2	41	3777	1.09
W3	24	2165	1.11
W4	40	2788	1.43
W5	46	2885	1.59
SP1	186	12509	1.49
SP2	0	1281	0
SP3	41	7092	0.58
SP4	80	9660	0.83
SP5	42	4821	0.87

The number of Y chromosome positive cells in the tracheal epithelial lining of female mice irradiated and transplanted with male whole bone marrow (W1-5 and CW1-4) or SP cells (SP1-5) after 3 months. CW1-4 were undamaged animals, and SP1-5 and W1-5 were 1 week post polidocanol damage. Cells are scored by Y chromosome FISH and the number of cells counted using DAPI staining of nuclei.

## Phenotype of donor-derived cells

### CD45 status and widefield counts

**Figure 1:**
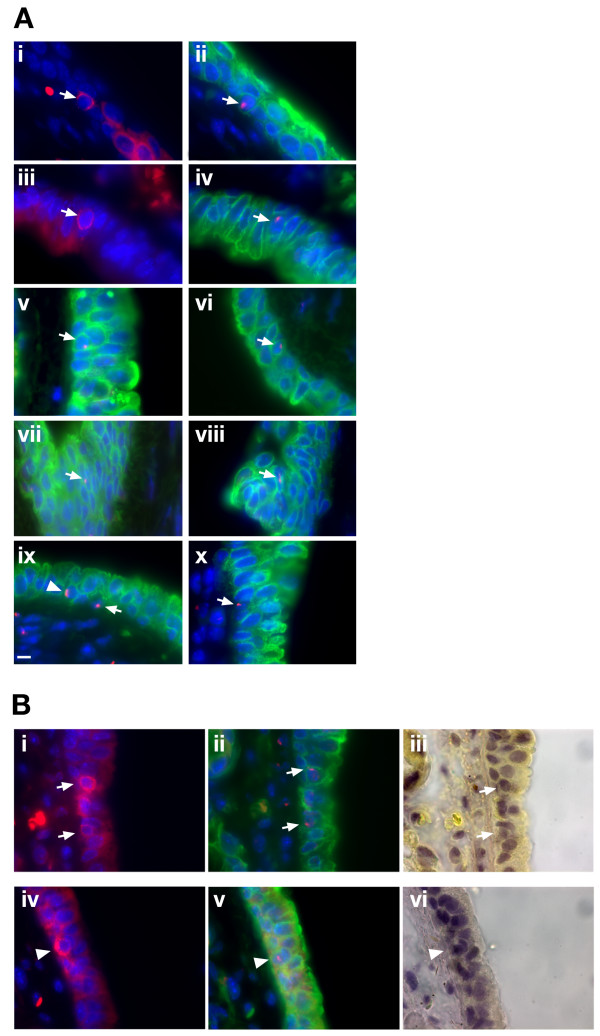
**Widefield visualisation of Y positive donor-derived cells and expression of CD45 and cytokeratin fall into four classes**. CD45 positive stained cells (CY3-red) were identified on tracheal sections, photographed, and their positions recorded. The CD45 signal was lost in subsequent manipulations. Sections were then subjected to Y chromosome FISH (Tx-Red) and immunohistochemistry for cytokeratin (FITC-green). Donor-derived (Y FISH positive) cells fell into four classes (Table 2). Figure 1A shows a CD45 positive (i) and cytokeratin positive (ii) coincident cell arrowed; CD45 positive (iii) and cytokeratin negative (iv) coincident cell arrowed; CD45 negative (not shown) and cytokeratin positive (v,vi,vii,viii); CD45 negative (not shown) and cytokeratin negative (ix, x) arrowed cells. ix also contains a cytokeratin positive cell marked with an arrowhead. 1B top panel shows two CD45 positive (i) and cytokeratin positive (ii) cells and the brightfield staining of this tracheal section (iii) with cells marked with arrows; Figure 1B bottom panel shows a CD45 positive (iv), cytokeratin negative (v) cell and photomicrograph (vi) shows brightfield staining of the same region with the cell marked with arrowhead. Additional file 4 shows cytokeratin staining alone of tracheal sections and also cytokeratin staining after Y chromosome FISH illustrating that the distribution of cytokeratin staining is the same. Scale bar 5 uM.

Cells had previously been identified that were of male origin and were positive for the epithelial marker pan cytokeratin[1]. This mixture of antibodies recognises cytokeratins 1,4,5,6,8,10,13,18 and 19 and is positive on simple, cornifying and non-cornifying squamous epithelia and pseudostratified epithelia[19]. We wished to further characterise the donor cell contribution so we used a protocol that allowed detection of CD45 (the universal blood cell marker) and cytokeratin on the same slide. The CD45 antibody used reacts with both alloantigens and all isoforms of the CD45 leukocyte common antigen. Sections from the trachea were exposed to the antibody protocol described in materials and methods. Figure 1A(i,iii) illustrates CD45 positive cells that reside in the epithelial layer that lines the trachea. These positive cells were marked and the sections then subjected to Y chromosome FISH and immuno-staining for cytokeratin. The CD45 signal was lost when the sections were subjected to the FISH protocol but the stored images could be found again and compared against the FISH and cytokeratin staining. Figure 1A(i) reveals that some cells are CD45 positive and both Y chromosome and cytokeratin positive (Figure 1Aii). Some are CD45 positive (Figure 1Aiii) and cytokeratin negative (Figure 1Aiv). The numbers of cells that were Y FISH positive and either positive or negative for CD45 and cytokeratin or both are given in Table 2. The majority (40.9%) of the donor-derived cells are cytokeratin positive and CD45 negative (see Table 2 and Figure 1A(v,vi,vii,viii)). However 19.3% (Table 2) of cells are positive for both antibodies (eg. Figure 1A(i,ii)) It seems likely that these cells have arisen from cell fusion as detailed in kidney and liver [11] but could be due to transdifferentiation and loss of the CD45 signal. The 15.9% (Table 2) of CD45 positive, cytokeratin negative cells (eg. Fig 1Aiii,iv) are in keeping with an inflammatory cell phenotype and therefore unremarkable. Figure 1A(ix and x) show arrowed cells that are CD45 and cytokeratin negative and represent the 23.9% of cells observed with this phenotype (Table 2).

**Table 2:** CD45 and cytokeratin expression of the Y chromosome positive cells found in the mouse trachea.

**Cell phenotype**	**Number of cells**	**% of total Y+ Cells**
CD45+, Y+, Cytokeratin+	17 out of 88	19.32%
CD45-, Y+, Cytokeratin+	36 out of 88	40.91%
CD45+, Y+, Cytokeratin-	14 out of 88	15.91%
CD45-, Y+, Cytokeratin-	21 out of 88	23.86%

Numbers of cells positive for Y FISH, and/or CD45 and/or cytokeratin by immunohistochemistry in the tracheas of SP cell engrafted mice three months after irradiation and one week after detergent-induced damage.

Overall by this analysis 60.2% (54/88 cells) of the donor-derived cells residing in the tracheal epithelium were cytokeratin positive.

### Deconvolution microscopy for cytokeratin immunohistochemistry and Y chromosome FISH

Sections that had undergone Y chromosome FISH and cytokeratin antibody treatment were subjected to analysis using deconvolved microscopy to further confirm their colocalisation. 205 cells out of 22271 were scored as Y positive (0.92%). Two investigators scored cells independently and then detailed analysis was undertaken on 125 Y chromosome positive cells (see Table 3). Of these, 101 were assigned a definite identity and 64 were scored cytokeratin positive (63.4%) and 37 cytokeratin negative (36.6%) by both investigators (Table 3). The remaining cells did not receive a definite identity by both investigators so were excluded. These numbers are not significantly different from the number of cytokeratin positive and negative cells analysed by widefield microscopy (60.2% and 39.8% respectively).

**Table 3:** Contribution of Y chromosome bone marrow SP cells to female recipient tracheas assessed by deconvolution microscopy.

	**Number of positive cells**	**% cells**
Y positive cells counted	125	100
Cells analysed	101	
Y FISH +ve cytokeratin +ve	64	63.4%
Y FISH +ve Cytokeratin -ve	37	36.6%

Numbers of cells analysed are those cells with a definite identity assigned independently by two investigators.

**Figure 2:**
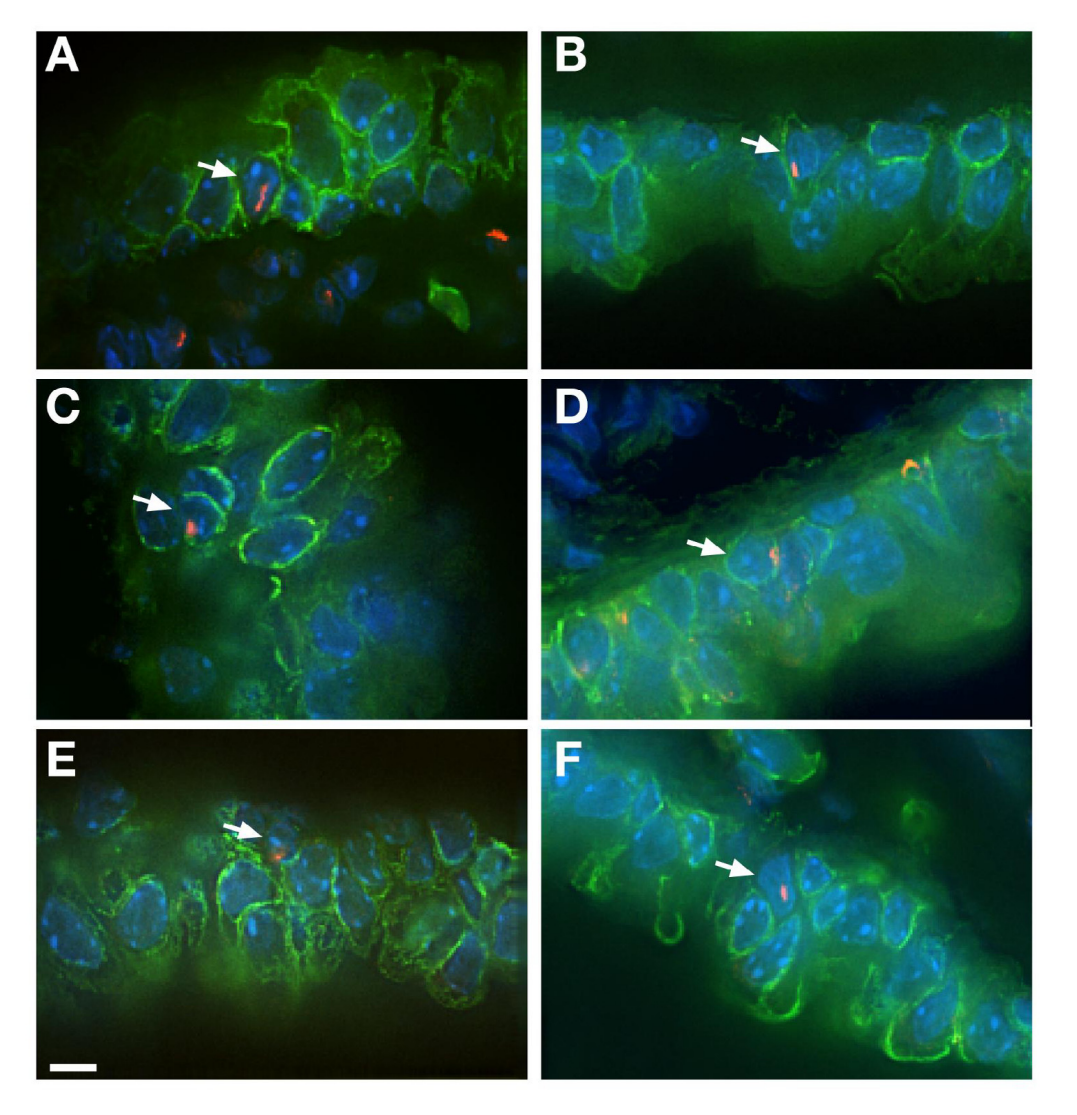
**Deconvolved visualisation of cells with Y chromosome FISH and cytokeratin immunohistochemistry**. Examples of Y chromosome positive cells by Y FISH (Tx-Red) and cytokeratin immunohistochemistry (FITC-green). Arrows mark cells positive for Y chromosome and either positive for cytokeratin (A,B,C,D) or negative (E and F). Videos of positive and negative cells can be found in additional files 2 (cytokeratin positive movie) and additional file 3 (cytokeratin negative movie).

Figure 2 shows a series of images that demonstrate denconvolved images of Y positive FISH cells and cytokeratin stained sections. A-D are positive for cytokeratin and E and F are negative. Movies of Y chromosome FISH positive cells that are either positive (additional file 2) or negative (additional file 3) for cytokeratin in additional files.

## Cytospin analysis

**Figure 3:**
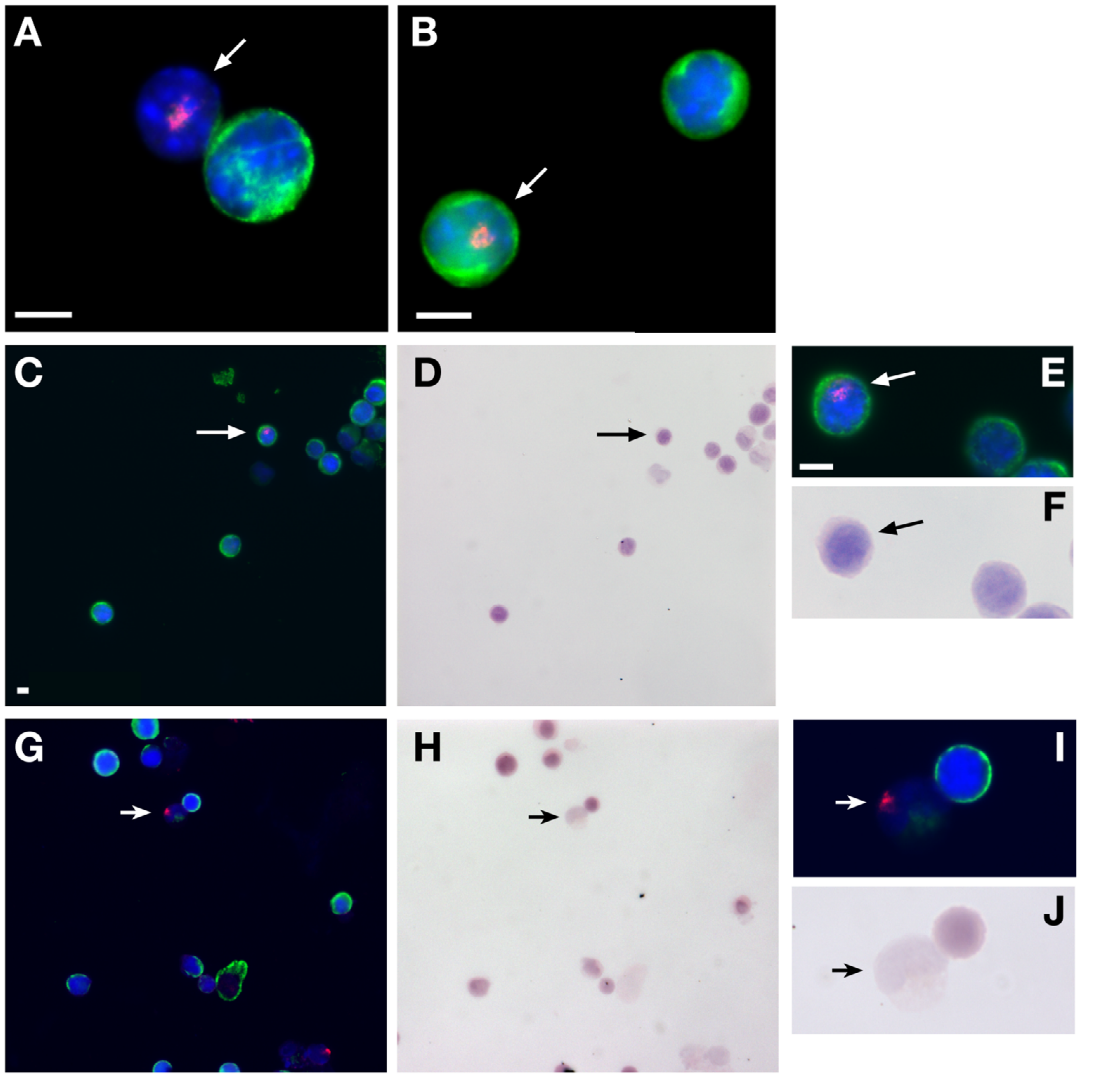
**Co-localisation of Y FISH and cytokeratin immunosignal in single cells**. Female animals engrafted with male bone marrow were sacrificed after three months and cells dissociated from the tracheas were cytospun onto slides and subjected to Y FISH (red) and cytokeratin immunocytochemistry (green). Y positive (donor derived) cells are arrowed and both Y chromosome positive and cytokeratin negative (panels A,G and I) and Y positive, cytokeratin positive cells (panel B,C and E) were observed. Non-arrowed cells in panels A and B are cytokeratin positive but not Y FISH positive and presumably host female derived. Panels C and G show donor cells (arrow) stained for presence of Y chromosome and then the slides were counterstained with H and E (panel D and H respectively). Panels E and I show the donor Y FISH positive cells (arrowed) with panels F and J showing the same cells stained with H and E. The donor cell (arrowed) in panels I and J is cytokeratin negative and has macrophage morphology.

The colocalisation of Y chromosome FISH positivity and cytokeratin immunosignal was further confirmed by taking tracheal cell preparations from the 3 month engrafted whole marrow transplanted mice. Following damage all the 5 animals (W1-5) tested showed evidence of Y positive cells by FISH carried out on the cytospin preparations and Figure 3 clearly shows examples of cells that are Y FISH positive and either cytokeratin negative (panels A, G and I) or cytokeratin positive (panel B, C and E). Following H and E staining, it would appear that the donor cell visible in panels G and I is a macrophage (panel J).

### Cytokeratin expression in the SPcells

**Figure 4:**
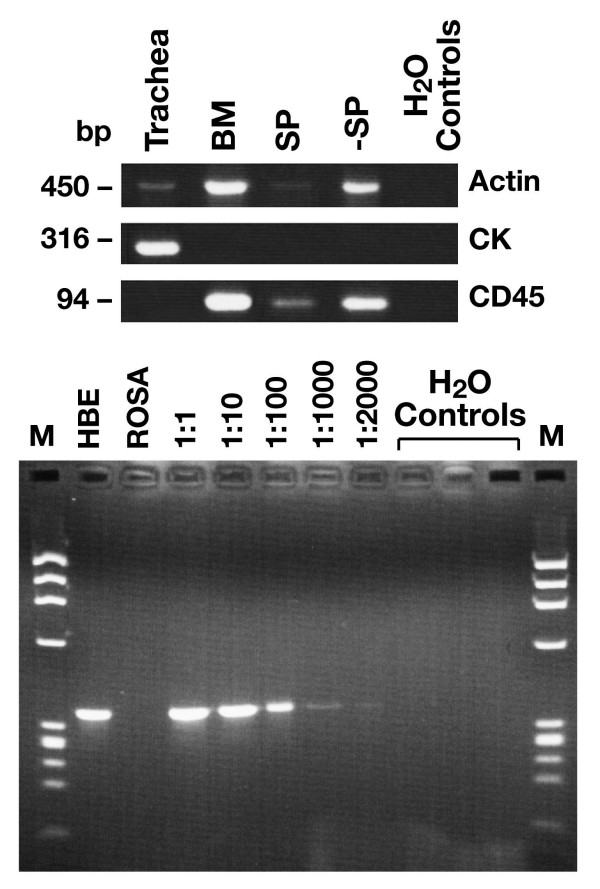
**Analysis of cytokeratin expression pattern in bone marrow and tracheal epithelial cells**. Reverse transcriptase PCRs for actin, CD45 or cytokeratin (CK) were carried out on RNA extracted from various samples. Cells were dissociated from adult mouse trachea, adult bone marrow (BM), side population cells isolated from BM as described[1](SP), bone marrow from which the SP has been removed (-SP). Equal numbers of cells were used for the RNA extractions in each case. No cytokeratin signal was detectable in the SP cytokeratin RT-PCR. The lower panel shows cytokeratin RT-PCR on human bronchial epithelial (HBE) cell RNA and ROSA (Rosa 26 bone marrow cells) as positive and negative controls respectively. The dilution series (where the ratio of HBE to Rosa 26 marrow cells is varied) indicates that the cytokeratin signal can be amplified and visualised using ethidium bromide from an RNA sample extracted from 1 HBE cell seeded into 2000 ROSA bone marrow cells. Greater sensitivity is obtained by blotting and probing with an internal oligonucleotide (data not shown).

We wished to eliminate the possibility that the donor-derived cytokeratin positive cells present in the tracheal epithelium had been present in the cells prior to injection into the animals. RT-PCR was carried out on RNA extracted from equal numbers of tracheal cells, whole bone marrow, SP cells and non-SP marrow population to see if we could detect any cells that expressed cytokeratin 19. No cytokeratin signal was detected in total bone marrow cells or in the SP or non-SP sorted cells, although a robust signal was evident in the tracheal sample (figure 4). This assay is sensitive to 1 in 2000 (Fig 4 lower panel) by ethidium staining and more sensitive by blotting with an internal oligonucleotide (data not shown). We also stained whole bone marrow cytospins with a pan cytokeratin antibody and could not observe any cells that were positive (0/1546-data not shown).

We conclude that the cells in the trachea are unlikely to have arisen by trapping in the airway following intravenous delivery.

## Discussion

There is ongoing debate in the literature as to whether bone marrow cells have the ability to populate tissues and express tissue specific markers. The airway has been an organ of major interest and was one of the first where this was demonstrated [20]. The fact that cells can repopulate from a bone marrow source has been strongly debated but irrefutable evidence of the ability of bone marrow cells to acquire functional characteristics of tissue specific epithelia has been generated using the fumarylacetoacetate hydrolase (FAH) deficient mouse. This mouse exhibits both liver injury and renal Fanconi syndrome when the mice are not maintained on the drug 2-(2-nitro-4-trifluoro-methylbenzyol)-1,3 cyclohexanedione (NTBC). Seven months after irradiation and bone marrow reconstitution with genetically marked *fah *wild type haematopoietic stem cells, robust donor cell contribution to the liver was observed. These fairly rare events (50–200 distinct donor cell-derived nodules) had undergone clonal expansion to achieve oligoclonal repopulation of about 30–50% of the liver mass. Thus the low engraftment efficiency was enriched by selective outgrowth of the healthy cells. Recently Held et al have reported that transplanted bone marrow cells can replace up to 50% of the proximal tubular epithelium[11]. However, once again the high level of repopulation required chronic and not acute injury and genetic selection.

Thus functional reconstitution can happen, but without the correct damage and selection it is a very inefficient process. Our data here supports this conclusion with airway repopulation observed only after detergent-induced damage. The damage agent used here was a detergent that strips the epithelial lining from the basement membrane. Following this treatment, no epithelial cells could be seen by histology[21] but basal cells may remain as described recently with naphthalene damage [22]. The mice are left for a week following the damage. This acute damage sequesters an inflammatory cell influx, which is largely resolved and a neo-epithelium is established by 7 days [1].

Our results show that using Y chromosome to detect donor-derived cells, between 0.6 and 1.5% of the epithelium is donor-derived in transplanted mice following damage. This level is comparable to the level reported for the distal lung[2]. We see 60% (table 2) of the donor-derived cells express cytokeratin. Cytokeratin is used as an epithelial marker and the tracheal epithelial cells are strongly positive for this marker. We do not detect any cytokeratin signal in bone marrow or SP cells by RT-PCR and so conclude that this must have been acquired *in vivo*. CD45 is a universal blood cell marker and is strongly positive on the SP cells we use for transplantation[14]. 32% of the cytokeratin positive donor-derived cells express both CD45 and cytokeratin. This could be due to fusion or transdifferentiation. The cells that are CD45 negative but cytokeratin positive have lost the CD45 marker which is expressed on all bone marrow-derived haematopoietic cells. Hepatocytes that have arisen from fusion with bone marrow-derived cells do not express CD45[7]. Harris et al report 0.2% of distal lung cells have a donor cell karyotype and they see no evidence for cell fusion in skin, liver or lung in their assay[2]. As the test for fusion is dependent on *cre *expression (which allows *lox *mediated excision and expression of a reporter gene) it can be difficult to be sure that a negative result is truly the absence of fusion rather than the lack of *cre *expression[23]. When we use male Rosa26 SP bone marrow cells as donors we see DNA evidence of the Y chromosome and Rosa 26 targeted allele in the tracheal epithelium of the female recipients, but we do not see expression of the β-galactosidase transgene carried by the Rosa26 mice[1]. This is unexpected as the Rosa26 promoter clearly does drive expression normally in the tracheal epithelia and other groups can use this marker to detect bone marrow-derived cells [24-26].

Recently Chang et al and Kotton et al reported experiments where they could not see any evidence of bone marrow cells reconstituting the lung epithelium following bone marrow transplant[5,8]. The first study used donor bone marrow cells expressing β-galactosidase either ubiquitously or under control of a type II pneumocyte-specific promoter. Using the cell type-specific promoter they saw no reporter gene expression and with the ubiquitous promoter the cells that appeared positive by widefield analysis were not supported when the analysis was repeated with deconvolution microscopy. Several aspects of our work are different to their experiments. The two most important being that we are studying the trachea rather than the distal airway and that we induce damage and repithelialisation of the trachea where they do not. Our findings are similar to those reported by other groups in that we see no bone marrow-derived engraftment unless the tissue is injured[6]. Chang et al rely entirely on radiation (5Gy split dose) induced damage but we do not find the level of irradiation we use (10.5 Gy single dose) to be sufficient to promote engraftment without additional epithelial damage. Additionally, Chang et al rely on expression of a donor-derived transgene to identify engrafted cells and yet (as mentioned previously) we have previously reported that the ubiquitously expressed β-galactosidase transgene is not a robust marker of transplanted cells in the airway[1]. Consequently, we use FISH for the Y chromosome as a marker of donor cells. We acknowledge their consideration that it is a necessity to use dual deconvolved microscopy and so we use this to confirm the co-localisation of Y FISH and cytokeratin expression.

The study by Kotton et al used whole bone marrow or SP cells to engraft irradiated mice and use a bleomycin damage protocol[5]. In contrast to their earlier reported work with mesenchymal bone marrow-derived cells [4], they see no evidence of engraftment in type II pneumocytes. The same comments about the reporter are relevant here. However this study used bleomycin as the damaging agent. Bleomycin is known to induce fibrosis[27]. This may mean that the type of donor bone marrow-derived cells engrafted into the lung would not be pneumocytes but fibroblasts[28]. These may be bone marrow-derived but would not be marked by the surfactant protein C promoter driven reporter Kotton et al use. Our use of the Y chromosome is less sensitive to epigenetic effects that may lead to shut down of gene expression. We show that 23.9% of donor-derived cells do not express CD45 or cytokeratin. It is possible that these cells may have fused with an endothelial or fibroblast cell and have subsequently lost their haematopoietic phenotype and CD45 expression.

Y chromsome FISH is a robust assay but may still underestimate the number of donor-derived cells. Our previous work demonstrates that we loose 13% of positive cells in male control 7 micron sections[1]. In addition Held et al[11] describe FAH recipient male mice that are transplanted with female wild type bone marrow cells and loose chromosomes from the donor cell. Cytospins of kidney cells show cells that are FAH positive (donor-derived) and yet have a normal recipient XY karyotype. They suggest that following fusion a mitotic reduction occurs, thus donor sex chromosomes are lost. If this occurred in the airway we would not detect all donor cells using Y FISH. The time between injury and sacrifice is fairly low (7 days) in our experiments so there may not be enough time for division and chromosome loss.

Rizvi et al recently reported evidence to suggest that bone marrow-derived cells could fuse with intestinal stem cells[29]. We only ever see isolated Y positive cells and so we do not see clonal expansion implying that there has been no involvement with a progenitor or somatic stem cell.

The possibility of using autologous bone marrow cells for genetic disease is an attractive prospect[30]. Combining gene therapy and bone marrow cell engraftment would bypass the need for immune suppression. The level of bone marrow engraftment that we describe here is around 1%, which is unlikely to achieve clinical benefit for a recessive disease such as cystic fibrosis. However, we have previously demonstrated using mouse models that 5% of normal levels of CFTR is sufficient to rescue the intestinal phenotype apparent in these animals[31]. Recently two groups have reported bone marrow transplantation of cystic fibrosis mice with wild type cells. Loi et al used naphthalene damage to injure the airway and saw only rare (0.02%) donor-derived cells expressing an epithelial marker[32]. The frequency of airway engraftment did not alter with or without damage which contrasts with our results and underlines the importance of the type of damage. Bruscia et al only found very rare cells that were donor-derived in both airway and gut[33]. They did not damage the tissue but the CF mouse gut is subject to continuous inflammation. This group also found very low levels of engraftment in both tissues (10^-5^) but could detect wild type CFTR mRNA. Surprisingly the bioelectric profile of mice transplanted with wildtype bone marrow was significantly improved in both gut and nose compared to those transplanted with bone marrow from cystic fibrosis mice. This implies that a very low level of cell therapy gives an amplified electrophysiological effect. An improvement above the very low level of engraftment observed by these two groups may have significant clinical impact. The improved level of engrafted donor cells observed in our study indicates that the type of damage is important and may lead to ways to manipulate cell therapy to a clinically relevant level. Identifying what signals induce efficient engraftment to the airway is essential to the clinical relevance of this work.

## Conclusion

This study demonstrates that following detergent-induced damage the airway of the re-epithelialised trachea contains cells derived from adult bone marrow cells. These donor bone marrow-derived cells are present in the epithelia at a frequency of around 1% and fall into four phenotypic classes based on expression of the epithelial marker cytokeratin and the universal blood cell marker CD45. The majority of cells express cytokeratin either with or without CD45. The transplant donor cells do not express cytokeratin so this novel phenotype must have been acquired *in vivo*.

## Competing interests

The author(s) declare that they have no competing interests.

## Authors' contributions

HM carried out the molecular genetic studies, immunoassays and participated in the study design and helped to draft the manuscript. PK carried out molecular and immunoassays. SW carried out immunoassays. CE carried out immunoassays. JD conceived of the study, and participated in its design and coordination and prepared the manuscript. All authors read and approved the final manuscript.

## Supplementary Material

Additional file 1 FDG conversion by bone marrow and peripheral blood cells from irradiated CBA and whole Rosa 26 bone marrow transplanted mice after 3 months. Granulocyte and lymphocyte populations in the marrow and peripheral blood from mice 3 months following lethal irradiation and transplantation with whole bone marrow. Samples from mice subjected to tracheal damage and undamaged controls. Click here for file

Additional file 2 movie of Y FISH positive, cytokeratin positive cell. Click here for file

Additional file 3 movie of Y FISH positive, cytokeratin negative cell. Click here for file

Additional file 4 Distribution of Cytokeratin. Immunohistochemical staining for cytokeratin (FITC – Green) shows that only the epithelial layer of the mouse tracheal sections stain with the antibody (Panels A and B). Male tracheal sections that have undergone Y chromosome FISH (TxRd – Red) followed by cytokeratin staining (Panels C and D) show the same distribution of cytokeratin staining as those stained with cytokeratin alone indicating that the FISH technique has not interfered with the distribution of cytokeratin staining. Magnification ×40. Click here for file
