# Supplementary material for: Following damage, the majority of bone marrow-derived airway cells express an epithelial marker
Source: Respir Res. 2006 Dec 19;7(1):145. doi: 10.1186/1465-9921-7-145 (PMC1764737; doi:10.1186/1465-9921-7-145)
Supplement: Additional file 1 — FDG conversion by bone marrow and peripheral blood cells from irradiated CBA and whole Rosa 26 bone marrow transplanted mice after 3 months. Granulocyte and lymphocyte populations in the marrow and peripheral blood from mice 3 months following lethal irradiation and transplantation with whole bone marrow. Samples from mice subjected to tracheal damage and undamaged controls. [file 1465-9921-7-145-S1.doc]

**FDG conversion by bone marrow and peripheral blood cells from irradiated CBA and whole Rosa 26 bone marrow transplanted mice after 3 months.**

**Marrow samples**

Undamaged controls


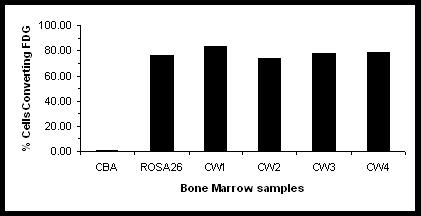


Lymphoid population

Undamaged controls


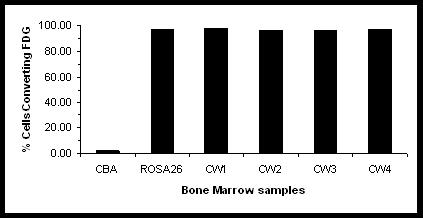


Granulocyte population

Lymphoid population

Trachael Damaged series


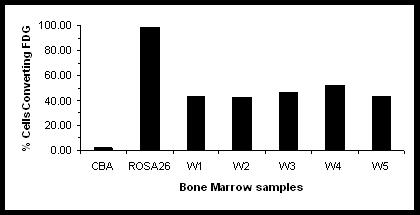


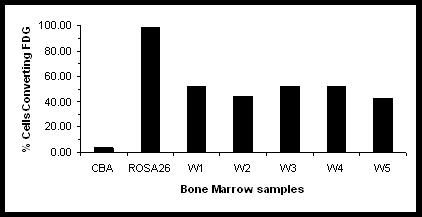


Granulocyte population

**Peripheral blood samples**

Undamaged controls

Lymphoid population

Granulocyte population

Damaged series

Lymphoid population

Granulocyte population
