# Supplementary figures and images for: Following damage, the majority of bone marrow-derived airway cells express an epithelial marker
Source: Respir Res. 2006 Dec 19;7(1):145. doi: 10.1186/1465-9921-7-145 (PMC1764737; doi:10.1186/1465-9921-7-145)

## Slide 1
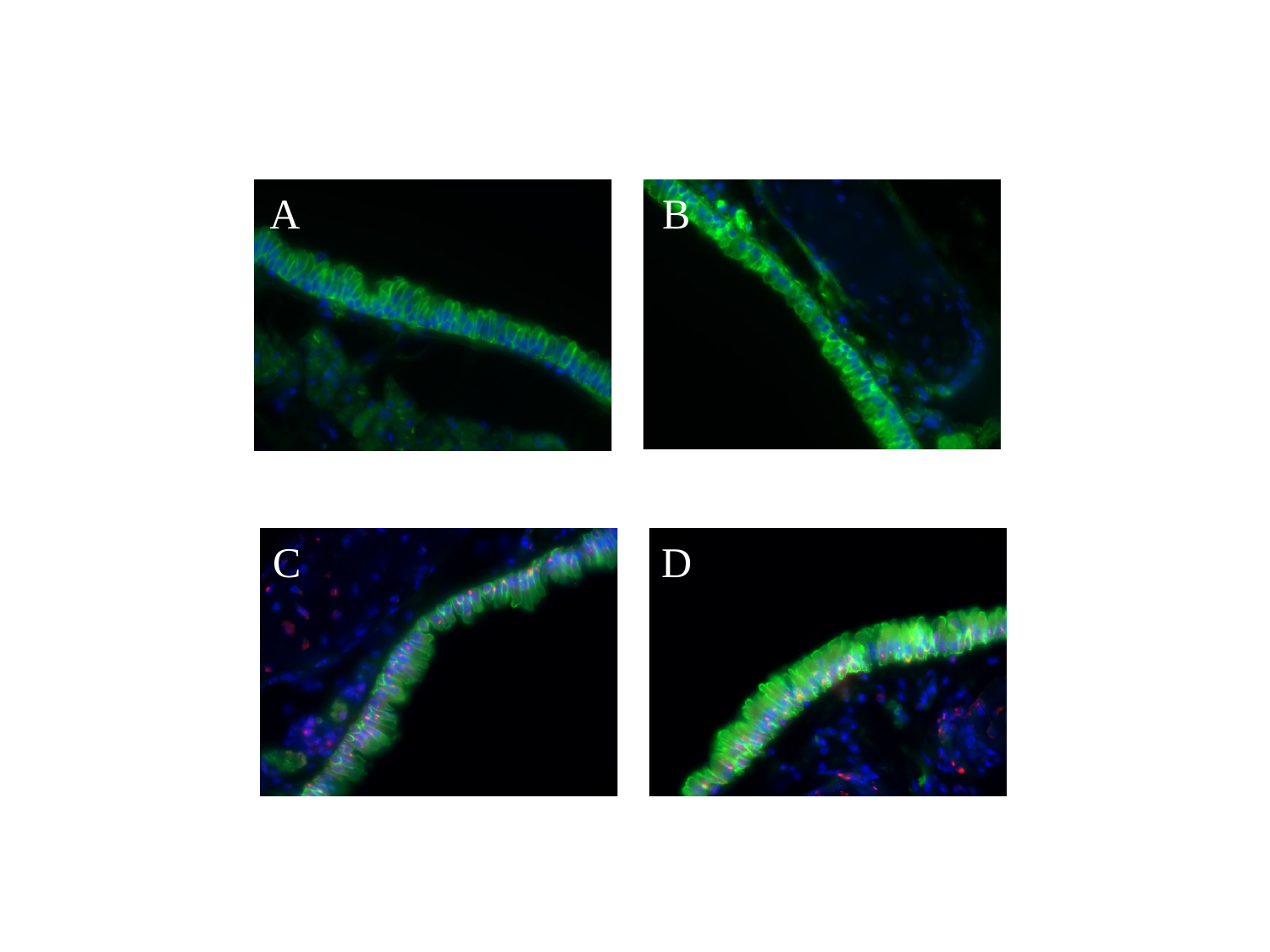

A
B
C
D

Supplement: Additional file 4 — Distribution of Cytokeratin. Immunohistochemical staining for cytokeratin (FITC – Green) shows that only the epithelial layer of the mouse tracheal sections stain with the antibody (Panels A and B). Male tracheal sections that have undergone Y chromosome FISH (TxRd – Red) followed by cytokeratin staining (Panels C and D) show the same distribution of cytokeratin staining as those stained with cytokeratin alone indicating that the FISH technique has not interfered with the distribution of cytokeratin staining. Magnification ×40. [file 1465-9921-7-145-S4.ppt]
